# The Shape of Data: a Theory of the Representation of Information in the Cerebellar Cortex

**DOI:** 10.1007/s12311-021-01352-6

**Published:** 2021-12-13

**Authors:** Mike Gilbert

**Affiliations:** School of Psychology, University of Birmingham, Birmingham, UK

**Keywords:** Theory, Cerebellum, Learning, Memory, Purkinje cells, Parallel fibres

## Abstract

This paper presents a model of rate coding in the cerebellar cortex. The pathway of input to output of the cerebellum forms an anatomically repeating, functionally modular network, whose basic wiring is preserved across vertebrate taxa. Each network is bisected centrally by a functionally defined cell group, a microzone, which forms part of the cerebellar circuit. Input to a network may be from tens of thousands of concurrently active mossy fibres. The model claims to quantify the conversion of input rates into the code received by a microzone. Recoding on entry converts input rates into an internal code which is homogenised in the functional equivalent of an imaginary plane, occupied by the centrally positioned microzone. Homogenised means the code exists in any random sample of parallel fibre signals over a minimum number. The nature of the code and the regimented architecture of the cerebellar cortex mean that the threshold can be represented by space so that the threshold can be met by the physical dimensions of the Purkinje cell dendritic arbour and planar interneuron networks. As a result, the whole population of a microzone receives the same code. This is part of a mechanism which orchestrates functionally indivisible behaviour of the cerebellar circuit and is necessary for coordinated control of the output cells of the circuit. In this model, fine control of Purkinje cells is by input rates to the system and not by learning so that it is in conflict with the for-years-dominant supervised learning model.

**Supplementary Information:**

The online version contains supplementary material available at 10.1007/s12311-021-01352-6.

## Introduction

Evidence of cooperative behaviour of functional cerebellar cell groups is technically difficult to obtain but is now emerging [1–6]. However, what form neurally coded information takes and how that orchestrates the behaviour of functionally grouped cells remains unknown. This paper builds on previous work [7, 8] to explain how the cerebellum converts input signals into internal code that controls the organised behaviour of functionally-grouped cell populations.

### The Proposal

The unspecialised cerebellar circuit has the problems (among others) of (1) handling eclectic inputs concurrently (tens of thousands of mossy fibres may drive input to a circuit at any moment), without (2) being adapted to discriminate (the response to individual signals does not depend on origin or data type). One of the issues around (1) is that ensemble firing contains more variables than single signals (number and fraction of active cells, spatial pattern, permutation and frequency distribution of rates, and so on). Not all variables are code. An effect of redundant variables must be controlled (in the sense that experimental variables are controlled, i.e, fixed or eliminated) in order to isolate the response to variables that are selected for an effect.

The proposed cerebellar solution is in two parts. The first is to code information in statistics present in ensemble firing of granule cells. Because the code is contained in statistics, it is insulated against an effect of other variables (at large numbers). For example, it is immaterial (in the model) which or how many input cells (mossy fibres) are active, or which granule cells they activate, or which granule cells fire at what rates, and so on. This frees the cerebellum to use intelligent (largely anatomical) adaptations of internal wiring to eliminate ‘bad’ variables.

The second part is to homogenise the code, meaning the same code is contained in any random sample of active parallel fibres over a numerical threshold. The regimented architecture of the cerebellar cortex means that the threshold can be represented by sagittal dimensions; this would explain the large size of the Purkinje cell dendritic arbour, for example. The sagittally aligned terminal branching pattern of mossy fibres, each branch ending in a cluster of terminals, is instrumental. As a result, all cells in a microzone receive the same code, so that control of the output cells of the circuit by Purkinje cells is coordinated.

This paper is one of a set which combine into a model of the cerebellar circuit. Circuit operation is not (in the model) governed by a central overarching principle. Instead, different parts of the circuit are adapted to solve different problems in different ways, although, functionally and anatomically, these are closely connected. Each paper covers a different aspect.

Stated briefly, the other papers so far have covered homeostatic regulation of granule cell activity [7] and ensemble memory, arguing that internal microcircuit wiring orchestrates a unitary response to remembered patterns, so that there is either a coordinated response of the whole circuit or there is no response at all [8]. The subject of the present paper is how mossy fibre rates are converted to granule cell rates and how information is represented in parallel fibre input to a microzone. While these elements combine into a functionally integrated framework, they do not cover all parts of the circuit or all aspects of function.

### Theoretical Context

The dominant theory of the cerebellum has for years been the supervised learning model [9–12]. Variants of the model share the central premise that learning teaches finely graded changes of parallel fibre transmission strength. In this way, learning modifies the Purkinje cell response to input in patterns that are received at modified synapses so that control by input rates is displaced by a learned response. The present proposals are in conflict with that idea. Interference by variable synaptic weights with the transmission of the rate code would impair control of Purkinje cell firing by the rate code and, in turn, control by Purkinje cells of nuclear cells, the output cells of the circuit.

The methodology is in some ways untypical of network models. Generally, network models make high-level assumptions and lack fine-grade detail. In artificial neural networks, cells are often represented as generic units. The present model, though it makes network-level proposals, is arrived at by collating the evidence at a detailed level and assembling it into a functionally coherent bigger picture so that the simulated network is populated with detail and cell diversity. High-level conclusions are drawn last. As a result, there is no top-down/bottom-up scale preference. Instead, the model is a physiologically detailed hypothesis which is computationally quantified by modelling.

It was contended in earlier work that parallel fibre activity contains two codes, a pattern code and a rate code [7], and that circuits are wired for a functionally indivisible response to the pattern code [8]. This paper makes the same case for the rate code, namely that the response to the rate code is also en bloc.

## Creating Order Out of Input Chaos

### Architecture of a Cerebellar Network

The pathway of input to output of the cerebellum forms an anatomically repeating, functionally modular network. Each network is bisected centrally by a functionally defined cell group, a microzone. The cerebellar cortex is divided anatomically seamlessly into these long and very thin regions, which form part of the modular cerebellar circuit, each containing several hundred Purkinje cells [2, 13–16]. Microzones lie at right angles across the path of parallel fibres, the axons of granule cells, which in turn receive input to the cerebellum from mossy fibres. Microzones form part of what are thought to be prevalently closed repeating circuits (Supplementary information, ‘A short review of the evidence of closed cerebellar circuits’). Parallel fibres pass orthogonally through the large sagittally flattened dendritic territory of Purkinje cells. Parallel fibres are so richly numerous that an estimated 350,000 pass through the territory of a single Purkinje cell [17, 18], of which around half make contact in passing.

Input to a microzone is to both of its long sides. Tens of millions of parallel fibres therefore pass through a microzone, originating in an area of the granular layer measuring, in the mediolateral direction, the maximum distance that granule cells have input from, and in the sagittal direction the length of a microzone they have input to. This is accordingly a field measuring around 6 mm [19, 20] × 15–20 mm [11, 13], respectively.

Tens of thousands (or more) of mossy fibres that innervate a network may be active at any time, in shifting, highly variable permutations of parameters – the number that are active, the combination they are active in, the termination pattern, the permutation of firing metrics, statistical properties of collective firing rates, and so on. The unspecialised cerebellar circuit does not discriminate between signals individually by origin or the nature of the information they represent – the cerebellum cannot ‘see’ upstream, at least in that detail.

Mossy fibres end in a cluster of glomeruli (each a ‘terminal’) [21, 22]. A mossy fibre axon may give rise to several terminal clusters, aligned sagittally, the direction of the long axis of a microzone [21–24], separated by a minimum distance. Clusters are estimated to average 7–8 terminals and to measure around 200 × 150 μm (sagittal × mediolateral) [24] – a ‘cluster field’. Cluster fields vary in number per cell and terminals per cluster and in size [21]. Fields are not discrete^1^ – terminals mix randomly with terminals of other mossy fibres, so mossy fibre signals are intimately and randomly mixed with other inputs to the same sagittal band.

Each terminal contacts a single dendrite of 10–100 granule cells [estimates vary: 25] so that a mossy fibre terminates in a sagittal band with divergence of 1:400–4000 (assuming 5 clusters/mossy fibre). Contact seems to be at random within a band [within coarse topographical strata: 26] so that granule cells randomly sample mossy fibre rates.

**Fig. 1:**
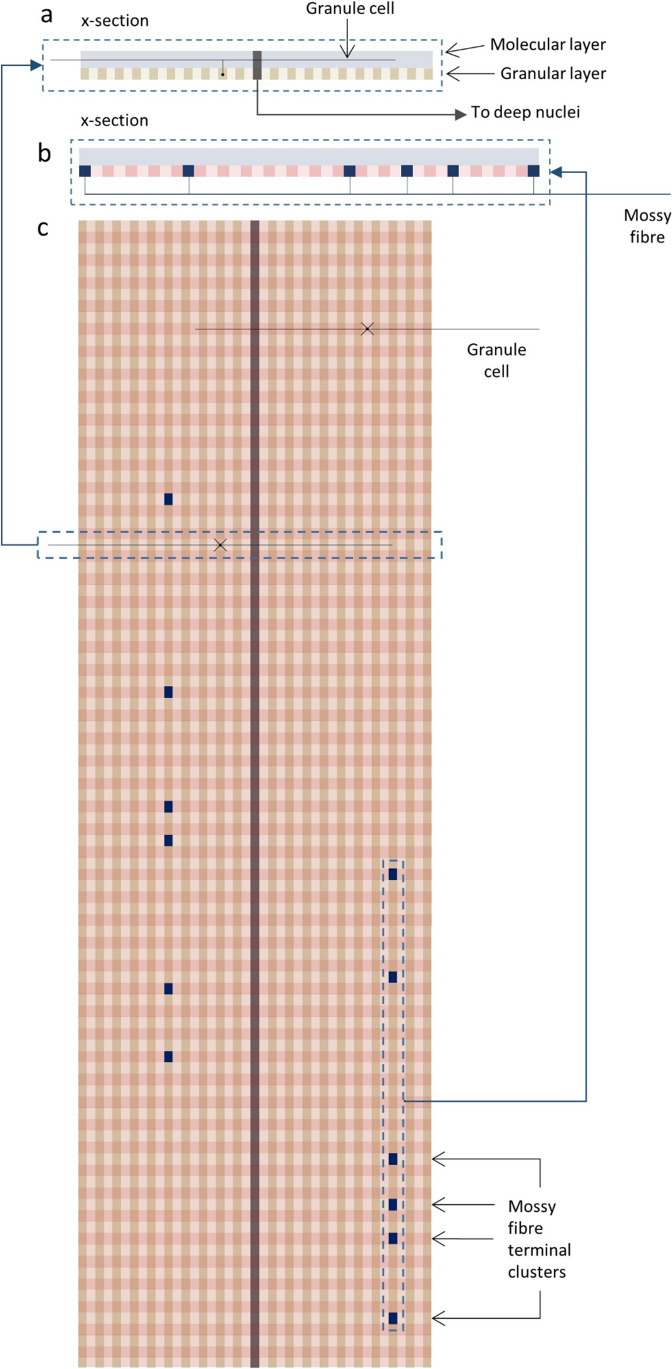
**Schematic of the region that supplies parallel fibre input to a microzone.** Curvature and folding of the cerebellar cortex are removed. (**a**) A beam (defined in the main text) shown in longitudinal cross-section (therefore at right angles to the view in (**c**)). Strictly speaking, a beam is a notional division of the granular layer; this view shows the molecular layer as well. The scale is around 10 × actual dimensions printed full page in A4 (actual size ~ 6 mm × 0.5 mm). A beam is crossed in the granular layer by 20 bands (as defined), therefore also appearing here in cross-section, shown in tan and light tan. Each band is the width of a microzone (assumed to be 150 µm). The central microzone is shown in dark grey. All relative dimensions are preserved except that the granule cell soma is 10 × relative dimensions (actual size 5 µm) and granule cell axon diameter is 100 × relative dimensions (actual size 0.1 µm). (**b**) Sagittal cross-section of part of a band (therefore also at right angles to the view in (**c**)) showing terminal branching of a mossy fibre, which approaches the cerebellar cortex from below and terminates in the granular layer as several sagittally aligned clusters of terminals. From this view, beams are in cross-section, shown in pink and pale pink. The scale is the same as (**a**) except mossy fibre axon diameter is 10 × relative dimensions (actual size 1 µm). (**c**) The region of the granular layer that supplies parallel fibre input to a microzone viewed from the cerebellar surface. Bands lie from top to bottom. Beams cross from side to side. The central microzone is shown in dark grey. Scale and relative dimensions are the same as (**a**) and (**b**) with the addition that granule cell dendrite length is 10 × relative dimensions (actual size ~ 13 µm). In reality, this region would contain something like 35 million granule cells and receive innervation from perhaps 400,000 mossy fibres

**Fig. 2:**
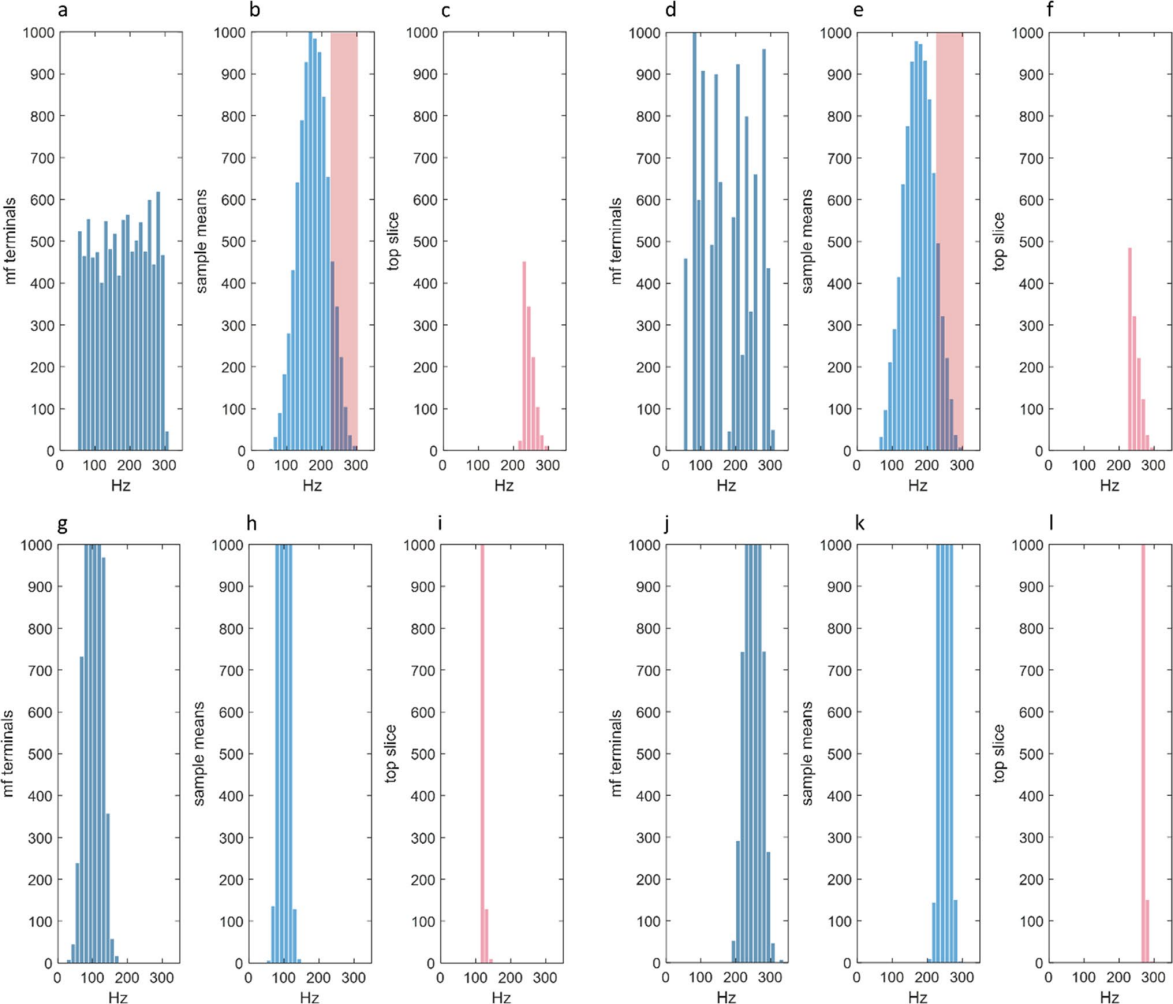
**The parallel fibre group rate code.** (**a**) Histogram representing mossy fibre input to a 200 µm-wide strip of granule cells that provides parallel fibre input to a Purkinje cell. The strip is divided into 40 fields. Each field receives a random number of mossy fibre signals in the range 1–30, each at a random frequency in the thought-to-be physiological range 50–300 Hz. (**b**) The distribution in (**a**) was sampled 4500 times, sample size 3, representing mossy fibre input rates received by the subset of granule cells in a beam which receive the minimum number of inputs needed to fire [7]. The sample means – the mean of each sample, representing the mean rate of input to each granule cell – are approximately normally distributed. The distribution of the sample means is always normal, by the central limit theorem, regardless of the distribution of the sampled population. (**c**) Not all 4500 granule cells fire. The number is regulated by inhibitory feedback and maintained at an average of around 1200. The pink data are the 1200 highest sample means – the top slice of the data in (**b**). The data in (**d**)–(**f**), (**g**)–(**i**) and (**j**)–(**l**) are generated in the same way. The only difference is the shape of the mossy fibre frequency distribution (but not the approximate number of mossy fibre signals). In (**d**), the number of signals in each bin is still randomly generated but with a variable bias, so that the distribution is uneven. In (**g**) and (**j**), the signal frequency range is narrower (SD 20) and randomly sampled from a normal distribution, but in different ranges – mean 100 Hz and 250 Hz, respectively. (Note that *y* axes in (**g**)–(**l**) are scaled to the data. Pink peaks in (**i**) and (**l**) would be off the (**c**) and (**f**) scale.) The mean of mossy fibre rates in (**g**) and (**j**) and of the top slice (**i**) and (**l**), respectively, have a linear relationship

**Fig. 3:**
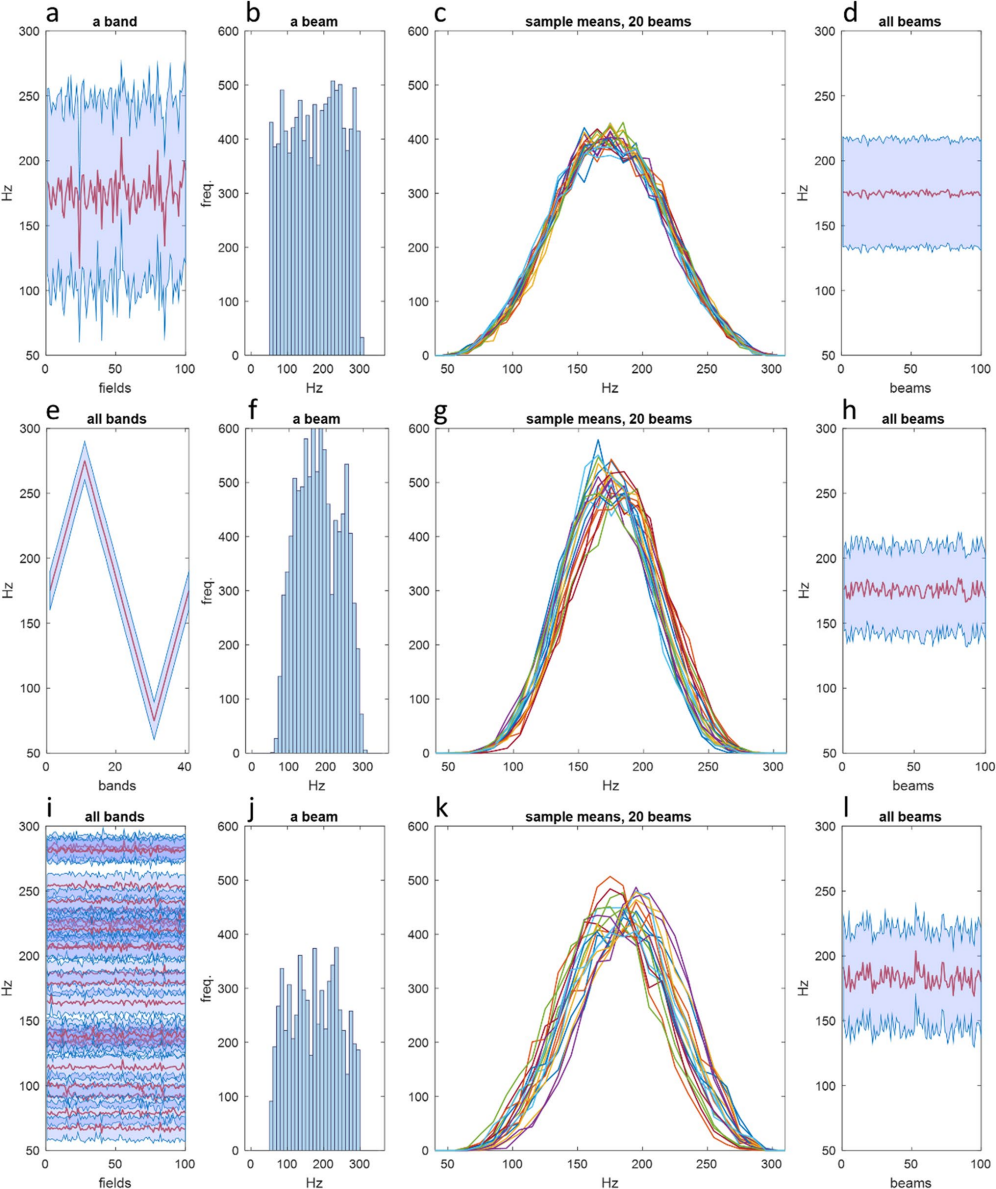
**Input disorder is translated into a homogenised internal code.** Simulation of recoding in the region supplying parallel fibre input to a microzone, divided into a matrix of 41 sagittal ‘bands’ crossed at right angles by 100 ‘beams’, forming 4100 sub-fields, each having the average dimensions of a mossy fibre terminal cluster. The number of mossy fibre inputs to each field is generated randomly, in the range 3–30% of mossy fibres active, adjusted for variation of the number of terminals that mossy fibres individually contribute to a field. The rates they fire at are sampled from input rates to a band as a whole. Three conditions were simulated which differ only in the limits of the range of otherwise randomly generated mossy fibre firing rates received by each band. In (**a**)–(**d**), all bands receive a random sample of rates in what is thought to be the physiological range of 50–300 Hz. (**a**) Mean and SD of input rates to each field in a single randomly selected band. (Note that this is a snapshot and does not show the passage of time.) (**b**) The frequency distribution of input rates to a single randomly selected beam. (**c**) Rates of input to each beam were sampled 4500 times, sample size 3, to represent random sampling by granule cells. The number of granule cells in a beam that receive input from 3 active mossy fibres was derived in Gilbert and Miall [7]. The sample means (the mean of each sample) was plotted as a line graph of a histogram. The distribution of the sample means is normal, by the central limit theorem. All beams have the same distribution (20 beams are shown, selected at random). (**d**) The mean and SD of the sample means in each beam. (**e**)–(**h**) Each band receives input at randomly generated rates in a fixed bandwidth (50 Hz) but with an oscillating range across bands, changing in steps of 10 Hz from band to band. (Note that (**e**) shows the mean and SD of input rates for each band, not each field as in (**a**)). (**i**)–(**l**) To stress test the system, input rates to each band are randomly generated in a fixed bandwidth (30 Hz) but randomly assigned range (lowest possible: 50–80 Hz; highest possible: 270–300 Hz). Each band has a one in two chance of receiving no mossy fibre input at all, so only around half of the bands receive input (19, shown in (**i**), which shows the mean and SD of input rates to each field for all bands). Only around 8% of mossy fibres are active, in a random pattern of active bands, each receiving input in a narrow and randomly selected range of rates. Nonetheless, high variance of input between bands is turned into, by contrast, relatively narrow variation of the distribution of the sample means (**k** and **l**)

If we divide a 6 mm × 20 mm region (the field providing parallel fibre input to a microzone) into cluster field-sized sub-regions, a sagittal row extending from side to side would be 75–100 cluster fields long (with approximately the footprint of a microzone) – which we might term a ‘band’ – and a row crossing it at right angles would be 40 cluster fields long – a ‘beam’. The region as a whole would be crossed by 40 bands and 100 beams, giving 4000 ‘squares’, each the area of a cluster field (Fig. 1). This organisation is part of the anatomical basis in evidence for the Fig. 3 model, but Fig. 1 also shows the Fig. 2 model in anatomical context. (There are 4100 squares in Fig. 1 to accommodate an extra band representing a central microzone.)

### Derivation of the Rate Code in a Single Beam

Granule cells have 3–5 dendrites, averaging 4, each receiving contact from a different mossy fibre. To take a mid-range estimate, a single mossy fibre terminal contacts what may be 50 granule cells [27, 28]. A minimum number of co-active mossy fibres is needed to make a granule cell fire – thought to be 3 [26, 29]. If we know the proportion of active mossy fires, $f$, which innervate a field, the number of granule cells which receive input to $k$ out of $n$ dendrites, out of a population, $N$, is a binomial function of $f$, $N$, $k$ and $n$. Accordingly, if we simulate mossy fibre activity by randomly generating (within specified limits), for each field, the fraction that is active, we can estimate the number of granule cells in each field that receive input from three or more active mossy fibres. The sum for all fields is the total in a beam [7].

If we now populate each field with input rates,^2^ randomly generated in the physiological range of mossy fibre signal frequencies or within some other constraints (Fig. 2a, d, g and j; the equivalent panels in Fig. 3 are 3b, f and j), we know the population of rates that is randomly sampled in a beam, the number of samples, and the sample size. The mean of random samples has a well-predicted distribution. If we take the mean of each sample, representing the mean rate of input to that granule cell, and plot the means as a frequency distribution, the sample means in a beam are invariably normally distributed, by the central limit theorem (Fig. 2b, e, h and k; 3c, g and k).

Input from at least 3 mossy fires is necessary but not alone sufficient for a granule cell to fire, as mossy fibre terminals also receive inhibitory feedback. Feedback limits firing to a subset of granule cells, regulating the number of parallel fibres which are active overhead at any time, keeping it in a narrow and stable range [7, 9, 29]. Therefore, granule cells that fire are a subset of a normal distribution. Assuming inhibition suppresses firing by granule cells that receive the weakest mean input rates, the distribution of the subset that fire has the shape of the upper end of the normally distributed sample means, the ‘top slice’ (Fig. 2c, f, i and l). The distribution of mean input rates to activated granule cells – those in the top slice – therefore has a highly constrained shape and an also highly constrained relationship with the distribution as a whole.

The mean of this distribution – termed the mean of the sample means – is the same as the mean of the sampled population, which is to say the mean of mossy fibre rates, again by the central limit theorem. It follows that the mean of the fixed-shape top slice has a narrowly constrained, and what may be a linear, relationship with the mean of mossy fibre rates. For example, if the shape of the frequency distribution of mossy fibre rates is roughly fixed, the mean of the top slice follows the mean of mossy fibre rates at a fixed distance (Fig. 2g–l).

The mossy fibre-granule cell connection is highly adapted in a range of ways for high fidelity transmission of mossy fibre rates [25, 30–34]. Assuming, as this would suggest, that granule cell rates are a relatively straightforward function of the mean rate of input to the cell, the mean and frequency distribution of granule cell rates are predicted by the range and shape of the top slice, and therefore upstream by mossy fibre rates.

A sagittal cross-section of the space over a simulated beam has about the same area as the Purkinje cell arbour so that granule cell activity in a beam provides parallel fibre input to a Purkinje cell that centrally bisects the beam. The output of this arrangement does not reflect only the subset of input rates received by granule cells that fire, or even the larger number that receive three (or more) inputs, but all mossy fibre rates received as input to a beam.

### Derivation of the Rate Code Received by a Microzone

Assuming mossy fibres terminate with a random distribution in a band, all fields in a band receive a random sample of rates. Accordingly, a beam randomly samples input rates to each of the bands it crosses. Beams that together provide parallel fibre input to a microzone each cross all the same bands, much as all files on a chessboard cross all the same ranks. As a result, we find the distribution of the sample means is the same, or very close to being the same, in all beams (Fig. 3c, g and k). That is, granule cell rates in all beams are generated by an almost identical top slice.

Nor is the pattern of active cells functionally different. Recoding in the granular layer ensures that the pattern of active parallel fibres overhead is randomly decorrelated [35] and that it is arbitrary how rates are distributed among active cells. That is, while the frequency distribution of granule cell rates is well predicted, it is arbitrary which cells fire and which cells fire at any particular rate. Contact by parallel fibres on a Purkinje cell is exclusively on dendritic spines on tertiary-most branchlets, one per spine [36]. Accordingly, variation between random patterns does not cause variation of the effect that is attributable to the site of contact.

This result is independent of (what are from a cerebellar view) chaotic input parameters, such as the number of mossy fibres that are active at any time, which ones, the shape of the frequency distribution of input rates, which cells fire at what rates, and so on.

In reality, beams are not discrete. Parallel fibre signals in a cross-section of the space overhead are not separate blocks of code. There are no boundaries or divisions in the code any more than there are anatomically between beams. Any volume of the same dimensions (which crosses the same bands) is interchangeable with any other. Put another way, information coded in parallel fibre signals is boundless in the sagittal plane. Any two beams which cross all the same bands contain the same code (where they are both bisected by the same sagittal plane).

Boundless is not quite right because this outcome is subject to a scale threshold, a lower numerical boundary – the number of parallel fibre signals below which statistical properties of group activity are no longer reliable. Because of the regimented architecture of the cerebellar cortex, this threshold has physical dimensions (in the sagittal plane again), assuming that active cells are a random sample of the population and firing rates are randomly distributed among active cells. As noted, these conditions are both met because they are properties conferred on parallel fibre group activity by decorrelation, itself a property of recoding [35].

It is unnecessary for Purkinje cells to receive all (or, strictly speaking, any) of the same signals in order to receive the same code. It is only necessary that they receive a representative sample, that is to say, a random sample that contains enough signals to meet the scale threshold – presumably a reason for the roomy dimensions of the Purkinje cell arbour. Around one in two parallel fibres that pass through a Purkinje cell territory make contact. (This also adds a second layer of randomisation, in addition to decorrelation.) But even if only a small fraction of parallel fibres are active, hundreds still make contact. With just 0.343% active, for example [an estimate derived in 7], a Purkinje cell receives contact from some 600 active cells.

To put this in other words, the parallel fibre group code received by functionally grouped Purkinje cells is homogenised – all Purkinje cells, anywhere in a microzone, receive the same code. Microzones are thin, only a few cells wide, as a practical near-equivalent of occupying the same plane. If microzones were wider or irregularly shaped, different parts of a microzone would receive a different code because they would receive parallel fibre innervation from (at least some) different bands. Of course, *any* width has this issue. But narrow width compared to parallel fibre length means that only a low fraction of parallel fibres that enter a microzone do not fully traverse it.^3^ Assuming a microzone span of 150 µm and a parallel fibre range of 3 mm in both directions from the cell body [19, 20], only about 2.5% of parallel fibres that enter a microzone do not fully traverse it.

There would also be timing issues with a wider or irregularly shaped microzone because different locations of a microzone would receive, at the same time, a different set of convergent signals, approaching from both sides. The problem would be aggravated because Parallel fibres are unmyelinated and very thin, and therefore very slow transmitters, perhaps the slowest in the brain [37].

In a sentence, the matrix-like organisation of the cerebellar cortex is designed to allow all Purkinje cells in a microzone to receive a homogenised rate code, with a precise and well-regulated relationship to the mean of mossy fibre input rates to the system, controlled for other variables.

### Digital to Analogue Conversion

This section makes a short physiological argument that the granule cell code has an analogue effect on individual Purkinje cells. This is a simple idea that came about by wondering how coding would be impacted by the fact that signals do not occupy a point in time but have a duration.

The small bore and slow transmission times of parallel fibres make them ‘probably the slowest … in the whole brain’ [37 p.41]. Transmission delay along a 2–3 mm parallel fibre branch is ~ 10 ms (or less) [37]. Granule cells typically fire in bursts, although spiking is variable between individual cells (some fire continuously, for example, in self-paced locomotion in mice [38]). The length of bursts varies: 10–20 ms has been reported in adult cats [26] and 8–40 ms in rabbits [39]. Bursts, accordingly, are usually longer than transmission time. It follows that for at least part of the duration of a burst, the entire length of the parallel fibre is active. For example, in a 25 ms burst, during the first 10 ms the active portion of the axon spreads outwards from the soma until the whole axon is active. That lasts for 5 ms. Then during the last 10 ms, the cell returns to its resting state, which also spreads out from the soma.

Functionally, the timing of a signal seems to be its arrival time, although evidence remains sparse. High-frequency bursts cause short-lived facilitation of release probability within the first few spikes rapidly, followed by a reduction of neurotransmitter release [39]. Following training (with a protocol analogous to eyeblink conditioning using anaesthetised animals), the first couple of spikes of a learned stimulus are sufficient to elicit a full conditioned Purkinje cell response [40], with adaptive timing preserved.

As far as the author is aware, the exact individual timing of signals in a time window, like the spatial pattern, is randomised. If so, the rate code is not received in a synchronised salvo. ‘Pattern’ does not mean a volley but turnover. The rate of turnover is stable because it is maintained by internal homeostatic regulation [7]. To maintain parallel fibre activity at the regulated level in short bins, the rate of turnover is robust. As a result, targets receive new inputs at a stable refresh rate in a spatially and temporally random pattern.

By this proposed means, targets receive a smoothly changing average rate of input in a rolling time window. As we have seen, the average is well predicted by the average mossy fibre rate. The bandwidth and shape of the frequency distribution of parallel fibre rates are highly constrained, so the average codes the response. Thus, coding by granule cell ensembles converts the short granule cell burst signature into a smoothly modulated effect on Purkinje cells. At least, this is an argument that all the necessary components are in place for that to happen.

## Discussion

### Scope

This paper describes a feasible relationship of mossy fibre rates and granule cell rates and how information is represented in ensemble activity of parallel fibre activity received as input to a microzone. A homogenised code is able to explain how firing of microzone-grouped Purkinje cells is orchestrated, consistent with evidence that concerted firing of Purkinje cells correlates with behavioural metrics [1–6]. It does not (by any means) claim to be a complete model of the cerebellar cortex. Nor does it suggest any reason to think there are not also other codes. Simply, it attempts to reconcile a range of evidence by explaining it as a coherent strategy.

### Supervised Learning Models

The dominant model of the cerebellum has for years been the supervised learning model. The central premise, and a necessary assumption, of the supervised learning model is that the cerebellum effectively implements a learning algorithm such that, following training, input to a Purkinje cell in a remembered pattern is passed through a corresponding set of precision-modified parallel fibre synaptic weights [9–12]. This has the result that the naive Purkinje cell response is displaced by a ‘desired’, learned response which the pattern triggers. The supervised learning model has variants, but these share the idea that algorithm-controlled synaptic weight adjustments are used to modify the naive response to input rates.

But if this was true, and learning displaced control by rates, what function would rates have? Functional graduation of parallel fibre synaptic weights is an idea that originally came parcelled up with a prediction that climbing fibres would provide the trigger for synaptic modification [9, 41]. Early on, a synaptic filter appeared to receive support when the prediction of a role of climbing fibres in synaptic modification was confirmed [42]. But later evidence was problematic [43–46]. In fact, an estimated 80–85% of parallel fibre-Purkinje cell synapses are strongly depressed, to the extent that there is ‘no detectable somatic response’ to granule cell stimulation [47 p.9676]. This is consistent with a high estimate of ‘electrically silent’ synapses made by parallel fibres activated by cutaneous stimulation [48].^4^ While attempts have been made to explain away these findings [11, 12], another interpretation is that they do not support an incorrect prediction which has exceeded its useful life.

The problems around graduated weights disappear if weights are, in fact, not graduated. This is not a new idea but in fact predicted by Marr [41], who is sometimes incorrectly associated with graded weights, which were added by Albus [9]. In Marr’s model, a synapse was ‘either totally modified or totally unmodified’ [41 p. 456], so that transmission is either faithful or not allowed, or the collective functional equivalent (also the position in: [7, 8]). This would be fatal to the supervised learning model but would permit the output of the circuit to be controlled by a chain of rate codes.

If so, a homogenised parallel fibre code would solve an important problem which the supervised learning model does not address. This is that, in the large majority of circuits, Purkinje cell signals are not cerebellar output. Instead, the circuit includes a cell group in the deep nuclei which receives the output of a microzone and contains excitatory projection cells which carry the output of the circuit. Contact of Purkinje cells on nuclear cells is with a convergence of 30–50:1 and a divergence of 1:4–5 [49], without any so far known internal organisation [Bengtsson and Jorntell in 50 p. 663]. The supervised learning model does not explain how, with this arrangement, Purkinje cell rates control nuclear rates. To expand on that, it does not explain (or really address) how a nuclear cell would decode or process convergent input at different rates from what seems to be a random sample of Purkinje cells. It also does not explain how nuclear cells in a group, which all receive a different mixture of rates, would each receive the correct rates and fire together in a concerted way. In the present model, the homogenised parallel fibre code coordinates the firing of microzone-grouped Purkinje cells so that they behave as a functional unit. As a result, nuclear cells receive a synchronised Purkinje cell rate both individually and as a group. It is immaterial which sub-group of Purkinje cells has input to any particular nuclear cell.

The individual timing of Purkinje cell simple spikes is irregular, but a smooth curve emerges across a step cycle when spikes are counted in step-locked bins over multiple cycles (in the rat, for example [51 Fig. 2]). The output of a microzone is channelled down onto a nuclear group that is smaller by around an order of magnitude, and divergence of Purkinje cells onto nuclear cells further increases convergence onto nuclear cells individually. In principle, this would allow (and may be designed to exploit) the same averaging effect in a single cycle, so that nuclear cells receive inhibition at a smooth averaged rate in real time. There is, in this model, no need for the internal organisation of the output of a microzone, and random sampling by nuclear cells of Purkinje cells is functional.

Control of nuclear cells explained in this way is consistent with reports of concerted intra-group behaviour of Purkinje cells. For example, in the flocculus, the averaged firing rate of functionally grouped Purkinje cells (approximated by binning spikes across the population of recorded cells) has a linear, rapidly translated relationship with eye movement [3], with 3–5 ms temporal precision. Likewise, saccades in monkeys have been shown to be under the control of groups of Purkinje cells in the oculomotor vermis, clustered by directional tuning of their climbing fibre input [4, 5].

The present proposals are in part an attempt to accommodate evidence of coordinated firing by Purkinje cells and provide a mechanism for it. However, that is not the only evidence, and purely linear transmission may be oversimplifying. Other evidence is discussed in the final section.

### Linear Code?

As noted, the proposed form of the granule cell code suggests no reason to think neural code may not also have other forms. Different forms may co-exist in intimate proximity. Indeed, the author has argued previously that parallel fibre activity contains two codes, a pattern code as well as the rate code, both contained in the same activity but encoded in independent variables [7]. The form of the code dictates the effect, and this may be a common theme. Timing codes are an example, such as spike synchrony models which argue that synchronisation of Purkinje cell spikes controls the timing of the rate and timing of spikes in the cerebellar nuclei [49, 52].

This paper describes the code received by Purkinje cells but does not then go further to propose how the parallel fibre ensemble rate code is converted into the Purkinje cell firing rate. This has been an area of recent development in ideas. There are theoretical reasons (and evidence) that high-frequency oscillation of functionally grouped Purkinje cells may synchronise convergent input to the cerebellar nuclei [53, 54]. Purkinje cell dendritic signalling is incompletely understood. Purkinje cells linearly code the strength of direct parallel fibre synaptic input both at single cell and ensemble level [4, 38, 55–58]. Modelling with a simulated Purkinje cell suggests that linear coding may be displaced by dendritic spikes in response to strong input at an adjustable threshold that depends on branch excitability and, in turn, on several factors that affect branch excitability [59]. Clustered patterns of parallel fibre activity have been reported [60] – a surprise, since in theory, decorrelation [35] should have the effect of causing active cells to be evenly spread out – but with an unknown effect.

Also, transmission of the parallel fibre code and the effect on firing by Purkinje cells are potentially further affected in a range of ways. This includes long-term synaptic plasticity – long a proposed site of information storage [9, 12] – but can, in theory, be anything that might alter the response, ultimately by affecting firing of the target cell. Examples include short-term plasticity of Purkinje cell simple spike pauses [61], dendritic membrane potential and excitability states [62–65] and sensitivity of Purkinje cell dendritic calcium transients to stimulus metrics [66, 67].

Onward transmission of the parallel fibre code is beyond the present scope. That said, the author has previously argued that much of the design of cerebellar cells and networks is to block or normalise an effect of most variables in order to isolate the effect of system-selected parameters [7], to control for unwanted effects. The rationale is that this ring-fences faithful transmission of the rate code by eliminating noise (which would otherwise cause system failure). The main strength of this idea is that it simplifies the picture – otherwise, there are too many variables to explain how they are all functionally integrated. The theoretical justification is that this is exactly what the cerebellum is itself highly adapted to do. Still, this does not fully explain control – or include an effect – of conditions that are reported to affect Purkinje cell dendritic transmission, referenced above, suggesting that the idea of a purely linear code controlled for other variables may need adjustment (or adding to).

## Supplementary Information

Below is the link to the electronic supplementary material.

Supplementary file1 (DOCX 38.4 KB)

## Footnotes

1 In theory, two fields could occupy exactly the same volume.

2 Adjusting for the fact that mossy fibres which innervate a field contribute a variable number of terminals.

3 A (previously proposed) reason that microzones are not even thinner is to ensure that only a modest region of unrecognised input (memory is discussed in the next section), perhaps only beam-sized, is sufficient to block output of the whole circuit: 8.Gilbert, M. and R.C. Miall, *Gating by functionally indivisible cerebellar circuits: a hypothesis.* The Cerebellum, 2021b. **20**(4): p. 518–532.

4 The same point is made in 8. Ibid.
