# Supplementary material for: The Shape of Data: a Theory of the Representation of Information in the Cerebellar Cortex
Source: Cerebellum. 2021 Dec 13;21(6):976–86. doi: 10.1007/s12311-021-01352-6 (PMC9596575; doi:10.1007/s12311-021-01352-6)
Supplement: Supplementary file 1 — Supplementary file1 (DOCX 38.4 KB) [file 12311_2021_1352_MOESM1_ESM.docx]

SUPPLEMENTARY MATERIALS

**A short review of the evidence of closed cerebellar circuits**

Most Purkinje cells that innervate a nuclear group originate in the same microzone or the same functional but dispersed group of microzones which form part of a multizonal circuit [1, 2]. Olivary cells contact Purkinje cells with which they co-terminate collaterally in deep nuclei [3] [see also 4, 5]. Collaterals are reciprocated by the inhibitory nucleo-olivary projection [6, 7], forming what are thought to be substantially closed circuits [5, 8-10]. Segregation is not perfect but organisation into circuits is well established. ‘CF [climbing fibre], olivonuclear, and corticonuclear axons project in a map-like fashion: neighbouring neurons in one region (olive, cortex, or deep nucleus) innervate neighbours in the other two regions’ [11 p.14]. In part of the C1 zone, for example, whose output is to the anterior interpositus nucleus and whose circuits control hind limb movements in rats, ‘a fine grain topography exists’ [12 p.16440].

Possibly, this does not hold for all circuits. Purkinje cell termination patterns are less focussed in the fastigial nucleus, for example [13]. Also, output of a microzone may be received by more than one nuclear location [2], probably as the result of Purkinje cell collateralisation [14]. Nonetheless, closed circuit organisation seems to be broadly preserved [2].

Deep nuclei integrate the descending input of Purkinje cells with collateral inputs from mossy fibres and climbing fibres to generate the output of the cerebellum [15, 16]. Contact by Purkinje cells on nuclear cells is preferentially somatic [17, p.3443] and individually strong [18], which may allow them to gate an effect of excitatory inputs, which are mainly (75%) dendritic [19]. However, not all mossy fibres send collateral projections to deep nuclei. Only 1 out of 15 mossy fibres originating in dorsal column nuclei, for example, had a collateral which terminated in a deep nucleus [20], and there is generally light collateral input to the dentate nucleus, such that the inhibitory input from Purkinje cells plays the primary role in controlling the dentate output [21].

Nucleo-cortical feedback is discussed in the main text. In addition to activation of the feedback pathway by disinhibition in the conditioned response, feedback cells may also receive collaterals of excitatory nuclear projection neurons which carry the main output of the circuit and which are disinhibited at the same time [22].

Evidence of group coded firing of functionally-grouped Purkinje cells is difficult to obtain but now emerging. In the flocculus, the averaged firing rate of functionally-grouped Purkinje cells (approximated by binning spikes across the population of recorded cells) has a linear, rapidly-translated relationship with eye movement [23], with 3-5 ms temporal precision. Likewise, activity of groups of Purkinje cells in the oculomotor vermis precisely matches the metrics of saccades [24, 25]. The significance (in this paper) is that control is rate coded in group activity, arguing for functionally unitary circuits. Rate control is supported by modelling [26].

REFERENCES

1. Apps, R. and M. Garwicz, *Anatomical and physiological foundations of cerebellar information processing.* Nat Rev Neurosci, 2005. **6**(4): p. 297-311.

2. Pantò, M.R., et al., *Corticonuclear projections of the cerebellum preserve both anteroposterior and mediolateral pairing patterns.* European Journal of Neuroscience, 2001. **13**(4): p. 694-708.

3. Llinás, R.R., K.D. Walton, and E.J. Lang, *7. Cerebellum*, in *The synaptic organization of the brain*, G.M. Shepherd, Editor. 2004, Oxford University Press: Oxford.

4. Ruigrok, T.J., *Cerebellar nuclei: the olivary connection.* Prog Brain Res, 1997. **114**: p. 167-92.

5. Bengtsson, F. and G. Hesslow, *Cerebellar control of the inferior olive.* Cerebellum, 2006. **5**(1): p. 7-14.

6. Hesslow, G. and M. Ivarsson, *Inhibition of the inferior olive during conditioned responses in the decerebrate ferret.* Exp Brain Res, 1996. **110**(1): p. 36-46.

7. Bengtsson, F., P. Svensson, and G. Hesslow, *Feedback control of Purkinje cell activity by the cerebello-olivary pathway.* Eur J Neurosci, 2004. **20**(11): p. 2999-3005.

8. Shinoda, Y., et al., *The entire trajectory of single climbing and mossy fibers in the cerebellar nuclei and cortex.* Prog Brain Res, 2000. **124**: p. 173-86.

9. Pijpers, A., J. Voogd, and T.J. Ruigrok, *Topography of olivo-cortico-nuclear modules in the intermediate cerebellum of the rat.* J Comp Neurol, 2005. **492**(2): p. 193-213.

10. Fujita, H. and I. Sugihara, *Branching patterns of olivocerebellar axons in relation to the compartmental organization of the cerebellum.* Front Neural Circuits, 2013. **7**: p. 3.

11. Ozden, I., et al., *Widespread state-dependent shifts in cerebellar activity in locomoting mice.* PLoS One, 2012. **7**(8): p. e42650.

12. Cerminara, N.L., et al., *Structural basis of cerebellar microcircuits in the rat.* J Neurosci, 2013. **33**(42): p. 16427-42.

13. Sugihara, I., et al., *Projection of reconstructed single Purkinje cell axons in relation to the cortical and nuclear aldolase C compartments of the rat cerebellum.* J Comp Neurol, 2009. **512**(2): p. 282-304.

14. De Zeeuw, C.I., et al., *Projections of individual Purkinje cells of identified zones in the flocculus to the vestibular and cerebellar nuclei in the rabbit.* J Comp Neurol, 1994. **349**(3): p. 428-47.

15. Bengtsson, F. and H. Jörntell, *Specific relationship between excitatory inputs and climbing fiber receptive fields in deep cerebellar nuclear neurons.* PLoS One, 2014. **9**(1): p. e84616.

16. Bengtsson, F., C.F. Ekerot, and H. Jorntell, *In vivo analysis of inhibitory synaptic inputs and rebounds in deep cerebellar nuclear neurons.* PLoS One, 2011. **6**(4): p. e18822.

17. Uusisaari, M. and E. De Schutter, *The mysterious microcircuitry of the cerebellar nuclei.* J Physiol, 2011. **589**(Pt 14): p. 3441-57.

18. Person, A.L. and I.M. Raman, *Purkinje neuron synchrony elicits time-locked spiking in the cerebellar nuclei.* Nature, 2012b. **481**(7382): p. 502-505.

19. de Zeeuw, C.I. and A.S. Berrebi, *Individual Purkinje cell axons terminate on both inhibitory and excitatory neurons in the cerebellar and vestibular nuclei.* Ann N Y Acad Sci, 1996. **781**: p. 607-10.

20. Quy, P.N., et al., *Projection patterns of single mossy fiber axons originating from the dorsal column nuclei mapped on the aldolase C compartments in the rat cerebellar cortex.* J Comp Neurol, 2011. **519**(5): p. 874-99.

21. Ishikawa, T., et al., *Releasing dentate nucleus cells from Purkinje cell inhibition generates output from the cerebrocerebellum.* PLoS One, 2014. **9**(10): p. e108774.

22. Svensson, P., F. Bengtsson, and G. Hesslow, *Cerebellar inhibition of inferior olivary transmission in the decerebrate ferret.* Exp Brain Res, 2006. **168**(1-2): p. 241-53.

23. Payne, H.L., et al., *Cerebellar Purkinje cells control eye movements with a rapid rate code that is invariant to spike irregularity.* Elife, 2019. **8**.

24. Herzfeld, D.J., et al., *Encoding of action by the Purkinje cells of the cerebellum.* Nature, 2015. **526**(7573): p. 439-42.

25. Herzfeld, D.J., et al., *Encoding of error and learning to correct that error by the Purkinje cells of the cerebellum.* Nat Neurosci, 2018. **21**(5): p. 736-743.

26. Abbasi, S., et al., *Robust transmission of rate coding in the inhibitory Purkinje cell to cerebellar nuclei pathway in awake mice.* PLoS computational biology, 2017. **13**(6): p. e1005578-e1005578.
